# Anaphylaxis and allergic reactions to COVID‐19 vaccines: A narrative review of characteristics and potential obstacles on achieving herd immunity

**DOI:** 10.1002/hsr2.787

**Published:** 2022-08-24

**Authors:** Sara Mahdiabadi, Nima Rezaei

**Affiliations:** ^1^ School of Medicine Tehran University of Medical Sciences Tehran Iran; ^2^ Network of Immunity in Infection, Malignancy and Autoimmunity (NIIMA), Universal Scientific Education and Research Network (USERN), Children's Medical Center Tehran Iran; ^3^ Research Center for Immunodeficiencies, Pediatrics Center of Excellence, Children's Medical Center Tehran University of Medical Sciences Tehran Iran; ^4^ Department of Immunology, School of Medicine Tehran University of Medical Sciences Tehran Iran

**Keywords:** allergic reaction, COVID‐19 vaccine, herd immunity, hypersensitivity, PEG, skin

## Abstract

**Background and Aims:**

Coronavirus disease 2019 (COVID‐19) is a highly contagious infection, and new variants of its causative virus continue to emerge all around the world. Meanwhile, mass vaccination represents a highly effective measure to reduce the disease burden. Not only do vaccines immunize individuals, but they also protect the entire population through achieving herd immunity. They are composed of various ingredients, some of which may induce hypersensitivity reactions, namely anaphylaxis and cutaneous allergic reactions. This review aims to provide an explicit overview of the pathophysiology, suspected responsible components, and management of COVID‐19 vaccine‐induced allergic reactions, and their effect on acquiring herd immunity.

**Methods:**

To perform this narrative review, a comprehensive literature search based on our selected terms was conducted in online databases of PubMed/Medline and Google Scholar for finding the relevant studies published from 2019 to 2022.

**Results:**

COVID‐19 vaccines introduce several advantages that outweigh their potential risks, such as allergic reactions. Allergic reactions are mainly attributed to polyethylene glycol and polysorbate excipients that can provoke IgE‐mediated reactions and hypersensitivity reactions. These reactions should be managed properly to avoid having serious sequelae.

**Conclusion:**

It is of great importance to immediately recognize and manage vaccine hypersensitivity reactions, especially anaphylaxis, to avoid allergic patients being excluded from the vaccination program, and more importantly, to stop the spreading of unfounded vaccine hesitancy leading to delayed herd immunity.

## 1 INTRODUCTION

The ongoing coronavirus disease 2019 (COVID‐19) pandemic induced by severe acute respiratory syndrome coronavirus 2 (SARS‐CoV‐2) has thus far infected nearly 220 million people and caused over 4.5 million deaths worldwide. Being highly contagious, COVID‐19 has led to substantial changes in our lives, so many countries have struggled to find the optimal strategy for combating it. None of these approaches that range from precautionary measures to searching for efficacious treatment options have been as promising as the vaccination strategy. Mass vaccination is an encouraging cost‐effective intervention to reduce morbidity and mortality, achieve herd immunity, and perhaps the most practical strategy to stop this pandemic.

Vaccination against COVID‐19 represents an opportunity to retrieve normal conditions of life. Vaccines help develop immunity by imitating an infection and training lymphocytic cells to fight that disease in case of future invasion.^1^ Not only do they immunize individuals, but they also protect the entire population by acquiring herd immunity. Vaccines have been applied as an effective public health intervention and have helped us dramatically improve human health and reduce the burden of infectious diseases since the mid‐20th century.^2^ However, as the body builds immunity, we can expect typical minor symptoms after vaccination, such as fever or fatigue.

Furthermore, allergic reactions to vaccine components are possible, albeit rare and without serious dangers. COVID‐19 vaccines are not an exception, as several allergic reactions to their injection have been reported. They are mainly attributed to polyethylene glycol (PEG) and polysorbate excipients that provoke immunoglobulin E (IgE)‐mediated reactions. Their clinical manifestations range from skin disorders to life‐threatening reactions like anaphylaxis, mainly in those with a history of allergies.^3^ Despite being rare and uncommon, these reactions have given rise to hesitation in receiving the COVID‐19 vaccine, which may delay achieving herd immunity towards this infection.

In this review, we provide an explicit overview of the pathophysiology of vaccine‐induced allergic reactions and the so‐far‐reported allergic reactions to available COVID‐19 vaccines. We also discuss their etiology, suspected responsible components, management, effect on vaccine hesitancy and aim to create a referable source for future studies. However, there is a limitation in comparing subcategories and different types of allergic reactions of each discussed vaccine in detail, as well as discussing allergic reactions to other available vaccines, both of which are due to the lack of sufficient clinical studies.

## 2 METHODS

To perform this narrative review, we conducted a comprehensive literature search based on the selected terms including “COVID‐19 vaccine,” “allergic reaction,” “hypersensitivity,” “herd immunity,” “anaphylaxis,” “cutaneous reaction,” “PEG,” and “polysorbate.” We explored online databases of PubMed/Medline and Google Scholar to find the relevant studies published from the emergence of COVID‐19 (2019) to 2022. We included the most appropriate studies without limiting them based on the article type. Preclinical studies were excluded.

## 3 BODY

### 3.1 Allergic reactions to vaccines

In recent years, there has been a surge in reports of possible allergic reactions to vaccination which stems from the increased use of vaccines in national health programs to avoid encountering newly emerged viral epidemics. However, the number of such confirmed cases is still relatively low, with an estimate of 1.31 cases of allergic reaction per million doses and less than 1 per 100,000 cases of anaphylaxis by different studies.^4^, ^5^ Yet, it is important to meticulously manage them to avoid severe complications. Influenza, measles‐mumps‐rubella (MMR), polio, and yellow fever vaccines are examples of immunization programs to which allergic reactions have been reported.

Vaccine‐induced reactions can be categorized in different ways. According to the World Health Organization (WHO), they can be categorized into systemic and local reactions.^6^ Systemic reactions mainly occur in the first hour after the injection and are infrequent, though it is important to recognize and manage them quickly. An anaphylactic hypersensitivity reaction is a prime example that can be either IgE‐mediated or non‐IgE‐mediated. Other examples include fever, skin rashes, delayed urticaria, malaise, muscle pain, headache, and diarrhea.^4^ However, local reactions appear as a result of postinjection inflammation and include pain, redness, and swelling at the injection site. Local reactions involve type IV hypersensitivity reactions such as subcutaneous nodules accompanied by itching and eczema and type III hypersensitivity (Arthus reactions).^4^

From another point of view, these reactions can be classified into acute and delayed categories. Acute or immediate‐type reactions are primarily type I hypersensitivity reactions that start within minutes up to 4 h of exposure to the relevant allergens, including some vaccine components.^5^ These reactions occur through the pathway of IgE‐mediated mast cell activation via Fcε receptor‐1, resulting in mast cell degranulation and secretion of histamine and other inflammatory markers. The events of this pathway have been confirmed by the specific IgEs detection and increased serum tryptase level.^7^ Vaccine excipients are considered more likely to cause these allergic reactions than the vaccine antigen and the residual nonhuman protein.^8^ It is of great importance to carefully evaluate acute‐onset IgE‐mediated hypersensitivity reactions since some of its symptoms, like anaphylaxis, may involve multiple organs rapidly and cause life‐threatening consequences. Such consequences can happen by means of Mas‐Related G Protein‐Coupled Receptor‐X2 (MRGPRX2), a receptor capable of inducing direct mast cell degranulation and anaphylactic reactions. This pathway is apparently more rapid, but more transient in comparison to IgE‐triggered events.^9^, ^10^ Other common symptoms of acute allergic reactions include: (1) urticarial rashes with itching and burning sensation, stemming from immunologic and nonimmunologic mast cell activation that usually resolve within 24 h and (2) angioedema with subcutaneous involvement that is often accompanied by pain and tenderness and generally resolves within 24–48 h.^11^

Delayed or type IV hypersensitivity reactions generally occur within 48 h after vaccination and reach their peak between 72 and 96 h.^5^ However, it has been stated that the initiation of symptoms may be delayed up to 2–3 weeks.^11^ These reactions are cell‐mediated and antibody‐independent. They occur as a result of T cell, macrophage, or monocyte overstimulation and cytokine release, leading to inflammation and tissue damage. Delayed reactions are usually self‐limiting and do not contraindicate the administration of future doses of the same vaccine. Although the aforementioned urticaria and angioedema generally happen in the context of acute reactions, they can also happen as delayed‐type reactions. In this case, they are a result of non‐IgE mediated processes such as complement system activation, leading to the generation of anaphylatoxins C1q, C3a C4, and C5a and Factor B, which consequently activate mast cells and trigger their degranulation.^12^, ^13^ Some delayed reactions, such as persistent hard nodules, may not have underlying immunologic pathogenesis. These nodules involve nonspecific inflammation or irritant reactions induced by adjuvants and are not necessarily due to immunologic hypersensitivity to vaccine constituents.^11^, ^14^, ^15^

### 3.2 COVID‐19 vaccines and allergic reactions

Exploration for finding an effective vaccine to arrest the COVID‐19 pandemic has resulted in the production of various vaccines with different modes of action that are mainly categorized into three groups. These include (1) vaccines using the whole disease‐causing virion, such as the Sinopharm and Covaxin vaccines; (2) adenoviral vector vaccines, such as Astra Zeneca, Johnson & Johnson, and Sputnik V COVID‐19 vaccines; and (3) messenger RNA (mRNA)‐based vaccines, prime examples of which are the Pfizer‐BioNTech and Moderna vaccines that use lipid nanoparticle (LNP) delivery system to prevent rapid enzymatic degradation of mRNA molecules.^16^

Thus far, allergic reaction reports to COVID‐19 vaccines include anaphylactic and cutaneous reactions.

#### 3.2.1 Anaphylaxis as a life‐threatening adverse reaction

Since the introduction of active vaccination against SARS‐CoV2 and the beginning of vaccination campaigns, early safety monitoring has detected anaphylactic reactions that emerge after the injection of the first dose vaccine and resolve after treatment.^17^ Anaphylaxis is a severe life‐threatening systemic hypersensitivity reaction that occurs due to mast cell degranulation and the widespread release of mediators such as histamine. With a rapid onset, it usually occurs within minutes after the exposure to a specific allergen and is accompanied by airway constriction, circulatory problems, low level of consciousness, and may be associated with skin and mucosal changes.^18^ Although anaphylactic reactions after vaccination are rare, they may be followed by serious complications. Thus, it is a matter of the utmost importance to identify and manage them quickly.

Most cases of anaphylactic reactions to COVID‐19 vaccines have occurred in less than 30 min after vaccination via immediate IgE‐mediated pathway in people with a history of allergic reactions, including anaphylaxis.^19^ However, as is the case with any other medication, it is possible that anaphylactic reactions resulting from vaccination happen in the absence of a history of allergic diseases.^20^

Regarding the epidemiology of these adverse events, reports imply that the overall occurrence of anaphylactic reactions due to COVID‐19 vaccination, estimated at around 4.5 in a million, is higher than the expected rate of severe allergic reactions with an incidence of one in a million.^13^, ^19^, ^21^, ^22^ Furthermore, studies demonstrate that the rate of these reactions in women is higher than men, one reason of which could be the greater number of women who have been vaccinated during these observations.^19^ Of note is that the incidence of adverse effects is lower with the Pfizer‐BioNTech than with the Moderna vaccine.^23^ A complete understanding of the underlying pathophysiology and the potential culprit of anaphylactic reactions to COVID‐19 vaccines is yet to be determined.^24^

#### 3.2.2 Cutaneous allergic reactions after administration of COVID‐19 vaccines

Another reported allergic reaction to COVID‐19 mRNA vaccines is dermatologic reactions,^25^, ^26^ some of which mimic the SARS‐CoV2 infection itself, suggesting that the skin eruptions may be caused by immune activation rather than directly caused by the virus.^27^ These reactions happen scarcely as a study reported their incidence 0.22% in vaccinated individuals and 16.54% of the whole vaccination adverse effects.^28^ Their wide spectrum involves injection site reactions and more extensive reactions. They include the more common delayed large local reactions, localized redness and swelling, urticaria, maculopapular rashes, and the less common erythromelalgia, chilblains, cosmetic filler reactions, and pityriasis‐rosea‐like eruptions. A study showed that delayed large local reactions and urticaria were the most common cutaneous reactions following vaccination with Moderna and Pfizer‐BioNTech, respectively.^29^ Post‐COVID vaccination facial swelling in people with previous use of cosmetic fillers was reported as a consequence of both mRNA vaccines.^29^, ^30^ It can be attributed to either immediate or delayed hypersensitivity reactions to filler ensuing from an immunogenic stimulus.^30^, ^31^, ^32^

Delayed localized cutaneous reactions to COVID‐19 in the injection site, called COVID arm or COVID vaccine arm, with a self‐limiting course have been observed in vaccinated individuals. These reactions emerge on average 7 days after the first dose injection. They can also appear after the second dose, though with a probability of less than 50%, faster development occurring in 2 days, and less severity.^29^ These reactions happening after 4 h of the injection do not inhibit receiving a second vaccine dose. Although immediate hypersensitivity reactions that occur less than 4 h after the injection, such as pruritus, flushing, angioedema, and urticaria, are contraindicated for second dose injection of the vaccine. Thus the importance of distinguishing immediate and delayed reactions lies in the clinical decision for the administration of the second dose.^33^

### 3.3 The culprit behind hypersensitivity reactions to COVID‐19 vaccines

Vaccines are comprised of two main kinds of components, namely the active component or the antigen and the additional components. The former can be the whole pathogen organism, parts of the organism, or inactivated toxins, and the latter can be conjugating agents, residual animal proteins, preservatives, metals, latex from sealing the vaccine ampoules, stabilizers, antimicrobial agents, adjuvants, contaminants, and culture medium used in the preparation process of the vaccines. Some of the most common individual vaccine components that may trigger anaphylaxis include egg protein, gelatin, milk protein, formaldehyde, thimerosal, and neomycin. Almost all components of the vaccine formulation can be considered as potential triggers for allergic reactions.^34^ However, active components seldom are the culprit, and hypersensitivity reactions, especially immediate and IgE‐mediated reactions, are usually traced back to the additives or excipients. Excipients are used to induce a stronger immune response, stabilize the potency of the vaccine during transportation or storage, prevent vaccine contamination, and for some other purposes.^12^ Being present in small amounts, these components generally do not induce allergic reactions. Nevertheless, In patients with unusually high levels of IgE antibody and those who have a genetic predisposition to produce significantly high amounts of antibodies during exposure to several allergens, severe reactions, including anaphylaxis, can originate from very small amounts of antigens and develop.^11^, ^35^ In spite of the fact that mRNA vaccines are new, most of their components were used in other medications and cosmetic products previously, which multiplies the odds of causing sensitization in genetically predisposed patients.^36^ Table 1 summarizes the main ingredients of mRNA‐ and vector‐based COVID‐19 vaccines as well as the incidence of anaphylactic reactions to each vaccine.^37^ Since currently, our understanding of the exact underlying pathogenesis is unclear, an investigation has been launched into the inciting agents to improve our knowledge about these reactions and their culprit.

**Table 1:** Vector‐ and mRNA‐based COVID‐19 vaccines’ ingredients and similar vaccines containing the same suspected allergen component

COVID‐19 vaccine	Active ingredient	Inactive ingredients	Storage	Incidence of anaphylactic reaction ^37^	Suspected allergen excipient	Other vaccine types with the same excipient	Predictive factors
Pfizer‐BioNTech	Nucleoside‐modified mRNA encoding the viral spike (S) glycoprotein of SARS‐CoV‐2	(4‐Hydroxybutyl)azanediyl)bis(hexane‐6,1‐diyl)bis(2‐hexyldecanoate)	−90 to −60°C	12.36/million	PEG 2000	N/A	previous anaphylactic reaction to drugs and vaccines; multiple allergies including drug allergies; mast cell disorders
		2[(PEG)‐2000]‐N,N‐ditetradecylacetamide					
		1,2‐Distearoyl‐sn‐glycero‐3‐phosphocholine					
		Cholesterol					
		Salts, sugars, and buffers					
Moderna	Nucleoside‐modified mRNA encoding the viral spike (S) glycoprotein of SARS‐CoV‐2	1,2‐Distearoyl‐sn‐glycero‐3‐phosphocholine	−20°C	20.39/million	PEG 2000		
		PEG 2000 dimyristoyl glycerol (DMG)					
		SM‐102					
		Cholesterol					
		Salts, sugars, and buffers					
AstraZeneca	Replication‐incompetent adenovirus vector, encoding a stabilized variant of the SARS‐CoV‐2 spike (S) protein	l‐histidine	2–8°C	17.64/million	Polysorbate 80	Influenza, HPV, Hepatitis B, DTaP, Rotavirus, zoster, meningococcal group B, Japanese encephalitis	
		l‐histidine hydrochloride monohydrate					
		Polysorbate 80					
		Salts, sugars, and buffers					
Johnson & Johnson	Replication‐incompetent adenovirus vector, encoding a stabilized variant of the SARS‐CoV‐2 spike (S) protein	2‐hydroxypropyl‐β‐cyclodextrin (HBCD)	−20°C	6.53/million	Polysorbate 80		
		Polysorbate 80					
		Salts, sugars, and buffers					

Abbreviations: N/A, not applicable; PEG, polyethylene glycol.

Polyethylene glycol (PEG), also known as macrogol, is a hydrophilic polyether polymer compound with variable chain length and a molecular weight that ranges from 20 to 10,000,000 g/mol. PEGylation, the process in which PEGs bind to the systemic drugs, not only increases molecular weight and prolongs circulation time but also prevents the opsonization of the drug by shielding it from the immune system.^38^ As PEGylation has been introduced as a technology that improves drug delivery, PEG is widely used as an excipient in various medications, cosmetics, and food products. Some of the pharmaceuticals that use this polymer‐based drug delivery system are laxatives, penicillin, about 30% of tablets, chemotherapy drugs, and many injectable formulations.^39^

In both Pfizer‐BioNTech and Moderna mRNA vaccines, one of the excipients is PEG 2000, a PEG with a molecular weight of 2000 g/mol. The modified mRNA used in these vaccines is easily taken up by mononuclear phagocytes, leading to rapid degradation by ribonucleases. Also, it has a poor penetration through the cell membrane originating from negative electric charge and high molecular weight. Thus, needing a protective shield for being delivered to cells, it is formulated into LNPs that contain low levels of PEGylated lipids, for stabilizing the nanoparticle and increasing their solubility by enabling the assembly of a hydrate shell.^40^ Consequently, the function of LNPs as nonviral vectors minimizes nanoparticle aggregation at the intramuscular and intradermal injection sites, improves nanoparticle spreading and drainage into the initial lymphatic vessels, facilitates cellular uptake by coating the mRNA with cationic lipids and lipids that mimic the phospholipids of the cell membrane.^20^, ^41^ After entering the cell, the mRNA encodes the viral spike protein and instigates the immune response against it.

It has been speculated that PEGylated lipids are the potential allergic components and the culprit agent for the aforementioned hypersensitivity reactions to COVID‐19 vaccines especially IgE‐mediated anaphylaxis. Although PEG‐containing products are generally considered safe, reports of IgE‐mediated allergic reactions and anaphylaxis to PEGs of different molecular weights have been described in the literature.^42^, ^43^, ^44^ There is difficulty in the assessment of anti‐PEG IgE via skin testing as wheal and flare are not always produced in patients with true PEG allergies.^24^, ^45^ Because of this, hypersensitivity to PEG has been described as rare previously, yet there has been a surge in reports of allergic reaction to PEG in recent years, which is attributed to the increased administration of certain drugs or certain personal hygiene products.^38^, ^46^, ^47^ A study has demonstrated that up to 70% of patients undergoing treatment with PEGylated products will develop anti‐PEG IgG antibodies.^48^ However, as mRNA vaccines use a novel technology and so far PEG has not been a common excipient in manufacturing vaccines, no reactions to the PEG‐containing vaccines have been described previously. An explanation for how a patient may be sensitized to PEG before COVID‐19 vaccination is their wide use in oral and injectable medications, cosmetics, and foods.^36^

Allergic reactions to LNPs happen in case of previous exposure and antibody formation against a component of the LNP. The only component toward which anti‐LNP antibodies have been detected in animal models and humans is the PEG polymer.^48^ The exact molecular mechanism of these reactions in humans is not clearly understood and may involve IgE‐ and non‐IgE‐mediated immediate hypersensitivity reactions.^49^ It has been proposed that although the existence of antibodies before COVID‐19 vaccination may lead to allergic reactions, they may also produce stronger immune responses and increase the vaccine efficacy. This can occur by increased dendritic cell uptake of the LNP, enhanced delivery of the mRNA to the cytoplasm, more viral spike protein expression, and improved capacity for presentation to T cells.^50^ There is a possibility that cross‐reactivity happens between PEG and polysorbate80, another excipient in some vaccines, due to containing chemically common structures.^43^

Polysorbate 80, also known as Tween 80, is another excipient that is contained in two vector‐based COVID‐19 vaccines, AstraZeneca and Johnson & Johnson. Polysorbate 80 is a PEG derivative and synthetic non‐ionic surfactant that is used in the food industry and medications, including 70% of injectable biological agents and monoclonal antibodies.^44^, ^51^ It constitutes a risk for allergic reactions to steroids and chemotherapeutics. Also, unlike PEG, polysorbate has been used in vaccines previously, and represents a rare cause of IgE‐mediated hypersensitivity reactions to vaccines. Thus, polysorbate 80 may cause allergic reactions after the injection of COVID‐19 first dose vaccine due to previous exposure and sensitization to this excipient.

### 3.4 Management and vaccination contraindications

Acute anaphylactic reactions occur without any strong correlation with age, sex, asthma, atopic status, or previous nonsevere reactions ^52^ and, in case of not being resolved, may cause severe sequels. Thus, it is necessary for all vaccination facilities to be equipped enough to immediately recognize and manage anaphylaxis.

It is of great importance to take the background allergic history of individuals thoroughly to determine whether COVID‐19 vaccination is contraindicated in them or not. One of the points to be aware of is a history of mild or severe allergic reactions, especially to PEG‐ or polysorbate‐containing products.^53^ It has been suggested that individuals with a history of allergic reactions due to any cause should be monitored for 30 min after COVID‐19 vaccination.^12^ Patients suffering from allergic skin diseases do not have an increased risk of anaphylactic reaction to the covid‐19 vaccine, and thus, vaccination should not be deferred in individuals with atopic dermatitis, urticaria, and other skin allergic reactions.^21^ Also, previous anaphylaxis to other vaccines, foods, and medical products are not contraindications of COVID‐19 vaccination. Instead, an immunoallergy workup should take place before the vaccination of these individuals.^21^, ^54^

All in all, according to the centers for disease control and prevention (CDC), COVID‐19 mRNA vaccines are contraindicated in case of a known history of a severe allergic reaction to any component of the vaccine.^33^ Thus, patients with a history of a severe allergy to COVID‐19 vaccine components, namely PEG or polysorbate, should avoid getting a COVID‐19 jab. Meanwhile, the second dose injection is contraindicated in the vaccine recipients who experienced a severe or immediate allergic reaction after the injection of the first dose.^33^ The former mainly includes anaphylactic reactions, while the latter includes allergic reactions that happen within 4 h after vaccination.^29^

When encountering the first signs of anaphylaxis, without any contraindication,^55^ it is urgent to immediately administer intramuscular epinephrine as the prime and life‐saving drug of choice to decreases mast cell degranulation. Subsequent actions include volume replacement, assessing vital signs, clearing airways, giving oxygen, administering short‐acting beta‐agonists in case of severe dyspnea, and nebulization with epinephrine in case of severe upper airway obstruction.^20^ For treating patients with cutaneous allergic reactions, symptomatic therapies, including topical corticosteroids, oral antihistamines, and pain‐relieving medications, are of use to resolve these reactions after a median of 3–4 days.^29^

### 3.5 The effect of allergic reactions to COVID‐19 vaccines on our journey to reach herd immunity

Vaccines are one of the most effective measures that curb the spread of viruses and strengthen our ability to combat epidemics. Identical to all medications, they may cause adverse events in some recipients. However, the advantages represented by the vaccines outweigh their potential risks, including hypersensitivity and ultimately anaphylactic reactions.

Mass immunization strategy by vaccination establishes vaccine‐induced herd immunity that, in addition to the particular vaccinated individuals, protects unvaccinated, immunocompromised, and vulnerable groups who are unable to develop immunity. This effect is exerted by the resistance of the society against a disease which itself stems from the immunity of a large proportion of the members and the consequent lower probability of an infected individual coming into contact with a susceptible person.^56^ Although achieving herd immunity through vaccination would considerably help control the disease, it will not end the pandemic.^57^ Eradication of the smallpox virus by global mass vaccination is a good showcase for vaccination‐induced herd immunity. In this respect, immunization of more than 80% of the global population against the virus decreased the number of susceptible hosts to lower than the threshold needed for transmission.^58^ The World Health Organization estimates that global vaccination programs save 2–3 million lives per year.^59^ It is assumed that for the induction of herd immunity against COVID‐19, a protective immunity rate of approximately 60% of the total population is required.^60^

**Figure 1:**
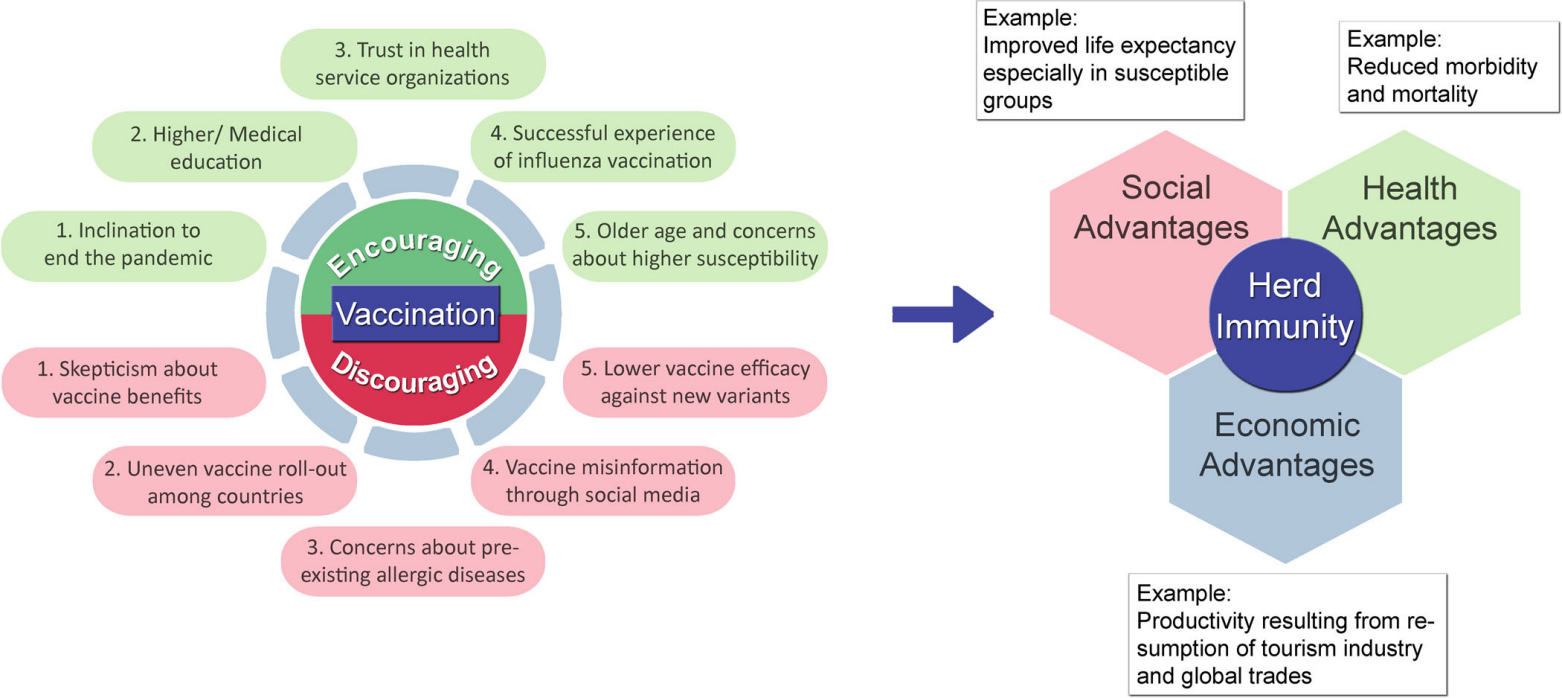
Key factors affecting vaccine acceptability and the journey to reach herd immunity. There are several positive and negative contributors to vaccine acceptability among people, an important factor of which is the history of pre‐existing allergic diseases and fear of severe reactions to vaccine components. The resultant vaccine acceptability rate determines the time of achieving herd immunity, the advantages of which involve social, health, and economic aspects

There are several factors that form the compliance of citizens with vaccination strategy and their confidence in vaccines. One of these factors is the public knowledge about the mechanism and benefits of achieving herd immunity.^61^ Other factors range from awareness in society about vaccines and their possible associated risks to religious, political, social, and economic status. Figure 1 shows the main factors that increase or decrease vaccine acceptability which has a strong association with herd immunity. Meanwhile, one of the most pivotal factors is the fear of hypersensitivity reactions peculiarly among those with a medical history of pre‐existing allergic diseases. Although allergic patients should not generally be excluded from the vaccination program, this may lead to a deep distrust of vaccines and delay deploying counter‐pandemic measures fast enough to balance out devastating economic, social, and public health consequences of the pandemic.

To avoid the detrimental repercussions of this unfounded vaccine hesitancy, health care workers should aim to dispel the misconceptions by providing accurate information for people about the potential benefits of being COVID‐19 vaccinated. Also, ensuring validity of vaccine‐related information through social networks is of great importance in eliminating conspiracy beliefs about vaccination.^62^ Moreover, to safely vaccinate susceptible individuals, they should be aware of the possible COVID‐19 vaccine hypersensitivity reactions and explicit protocols for managing them.

## 4 CONCLUSION

In summary, COVID‐19 vaccines introduce several advantages that outweigh their potential risks, such as allergic reactions that occur through different pathways and whose proper management can arrest severe outcomes. These allergic reactions include anaphylaxis, a severe life‐threatening systemic reaction due to mast cell degranulation and mediators' release, and cutaneous reactions including delayed large local reactions, urticaria, maculopapular rashes, chilblains, cosmetic filler reactions, pityriasis‐rosea‐like eruptions, etc. PEG and polysorbate 80, excipients that stabilize vaccine potency and help it induce a stronger immune response, can provoke IgE‐mediated reactions and are the main identified culprits for these hypersensitivity reactions.

COVID‐19 vaccines are not the first pharmaceutics to which allergic reactions have been reported. Previous allergic reactions to medications, vaccines, and cosmetic products with different excipients have been reported. Moreover, vaccine hypersensitivity reactions are rare and, if managed properly, do not result in serious sequelae. Thus, the pivotal point is to provide well‐founded information for the public to quash vaccine hesitancy and tackle one of the problems that decrease vaccine acceptability and disrupt achieving herd immunity.

## AUTHOR CONTRIBUTIONS

Conceptualization, Data Curation, Writing–Original Draft Preparation, Writing–Review and Editing: Sara Mahdiabadi. Conceptualization, Writing–Review and Editing, Supervision: Nima Rezaei. All authors have read and approved the final version of the manuscript. The corresponding author had full access to all of the data in this study and takes complete responsibility for the integrity of the data and the accuracy of the data analysis.

## CONFLICT OF INTEREST

The authors declare no conflict of interest.

## TRANSPARENCY STATEMENT

The lead author (Nima Rezaei) affirms that this manuscript is an honest, accurate, and transparent account of the study being reported; that no important aspects of the study have been omitted; and that any discrepancies from the study as planned (and, if relevant, registered) have been explained.
